# HIF and fumarate hydratase in renal cancer

**DOI:** 10.1038/sj.bjc.6603547

**Published:** 2007-01-09

**Authors:** S Sudarshan, W M Linehan, L Neckers

**Affiliations:** 1 Urologic Oncology Branch, Center for Cancer Research, National Cancer Institute, 10 Center Drive, Bldg 10 CRC Room 1-5940, Bethesda, MD 20892-1107, USA

**Keywords:** hereditary leiomyomatosis and renal cell cancer, kidney cancer, fumarate hydratase, hypoxia inducible factor

## Abstract

Hereditary leiomyomatosis and renal cell cancer is a recently described hereditary cancer syndrome in which affected individuals are predisposed to the development of leiomyomas of the skin and uterus. In addition, this clinical entity also can result in the development of biologically aggressive kidney cancer. Affected individuals harbour a germline mutation of the *fumarate hydratase* (*FH*) gene, which encodes an enzyme that catalyses conversion of fumarate to malate in the Kreb's cycle. Thus far, proposed mechanisms for carcinogeneis associated with this syndrome include aberrant apoptosis, oxidative stress, and pseudohypoxic drive. At this time, the majority of accumulating data support a role for pseudohypoxic drive in tumour development. The link between *FH* mutation and pseudohypoxic drive may reside in the biochemical alterations resulting from diminished/absent FH activity. These biochemical derangements may interfere with oxygen homeostasis and result in a cellular environment conducive to tumour formation.

Kidney cancers represent a heterogeneous group of malignancies linked by the primary site of pathology. Despite originating in the kidney, renal cell carcinoma (RCC) is a disease with varying genetic basis. Biologic characterisation of this disease has been greatly aided by the investigation of families with multiple members affected by RCC. Probably the earliest and best-characterised familial form of kidney cancer is von-Hippel Lindau (VHL). von-Hippel Lindau families are susceptible to the development of clear cell histology RCC owing to inherited alterations of the *VHL* gene (Latif *et al*, 1993). Moreover, the *VHL* gene has also been implicated in the development of a significant proportion of sporadic clear cell kidney cancers (Gnarra *et al*, 1994). In addition to *VHL*, other genes have been identified to be involved with inherited kidney cancer, for example, *c-met* in hereditary papillary renal carcinoma (Schmidt *et al*, 1997).

More recently, an inherited neoplastic syndrome has been identified and referred to as hereditary leiomyomatosis and renal cell cancer (HLRCC) (Launonen *et al*, 2001). Families affected by HLRCC possess a germline-inactivating mutation in the gene encoding the Kreb's cycle enzyme, fumarate hydratase (FH) (Tomlinson *et al*, 2002). These individuals are predisposed to development of benign leiomyomas of the skin and uterus as well as highly aggressive kidney cancers (Launonen *et al*, 2001; Tomlinson *et al*, 2002; Wei *et al*, 2006). Somatic genetic events inactivating the second *FH* allele in leiomyomas and kidney tumours have been identified (Tomlinson *et al*, 2002). Kidney cancers are less penetrant than leiomyomatous manifestations in HLRCC-affected families (Wei *et al*, 2006). Renal tumours have been identified in approximately one-third of HLRCC families evaluated at the National Cancer Institute; however, family recruitment criteria may have favoured selection for families affected by kidney cancer (Wei *et al*, 2006). Kidney tumours that develop in patients with HLRCC are biologically very aggressive. Of 13 individuals identified with kidney cancer in the first reported cohort of North American families, nine patients succumbed to metastatic disease within 5 years from initial diagnosis (Toro *et al*, 2003). As a result, regular screening of affected HLRCC individuals is advocated. Although *FH* mutation has been linked to the development of RCC in the context of HLRCC, its role in sporadic kidney cancers appears to be limited (Kiuru *et al*, 2002). Morris *et al* (2004) examined DNA from RCC samples. They were unable to detect *FH* mutations in all nine cell lines tested. Of the 46 tumour samples examined, no mutations could be identified in 42. In the remaining four tumour samples, the alteration identified was a silent single-nucleotide polymorphism (Morris *et al*, 2004). In addition, Kiuru *et al* (2002) were unable to identify *FH* mutations in 52 sporadic RCCs. These data suggest that biallelic loss of *FH* is necessary to promote HLRCC-associated renal tumorigenesis. As complete loss of FH activity is developmentally disfavoured owing to the severe energy deprivation imposed on the developing brain by such a genetic defect, appearance of HLRCC appears to be restricted to those otherwise healthy individuals hemizygous for *FH* in whom a somatic mutation has inactivated the second allele. Despite a potentially limited role for *FH* in sporadic RCC, investigation of the molecular basis of HLRCC provides a unique opportunity to identify signalling pathways that may be important for kidney cancer aetiology.

## ROLE OF FH IN KIDNEY CANCER

The role of *FH* alterations in the development of HLRCC is under intense investigation. As mentioned, FH is an enzyme of the Kreb's cycle that catalyses the conversion of fumarate to malate. It appears that *FH* acts as a tumour suppressor gene and fits Knudson's two-hit model (Tomlinson *et al*, 2002). Patients with HLRCC inherit a mutant copy of the *FH* gene as well as a wild-type copy. Tumour formation appears to occur when there is somatic alteration of the wild-type copy (Tomlinson *et al*, 2002). Although allelic loss appears to be the most common alteration, insertion and missense mutations have also been described in kidney tumours from patients with germline mutations of *FH* (Tomlinson *et al*, 2002).

Given the reliance of cells on the mitochondrion to generate adenosine triphosphate by oxidative phosphorylation, it seems that a cell with a defective tricarboxylic (TCA) cycle would be at a disadvantage. However, FH is not the only TCA enzyme whose inactivation is linked to neoplasia. Mutations of *succinate dehydrogenase* (*SDH*) have been implicated in the development of a cancer syndrome referred to as hereditary paraganglioma and pheochromocytoma (HPGL). Succinate dehydrogenase catalyses the conversion of succinate to fumarate. In contrast to FH, which functions as a homotetramer (Wei *et al*, 2006), SDH consists of four different subunits, named A, B, C, and D (Gottlieb and Tomlinson, 2005). Mutations of *SDH-B, SDH-C*, and *SDH-D* have all been identified with HPGL (Baysal *et al*, 2000; Niemann and Muller, 2000; Astuti *et al*, 2001).

Postulated mechanisms for these inherited cancer syndromes include aberrant apoptosis, oxidative stress, and pseudohypoxic drive (Gottlieb and Tomlinson, 2005). Of these, pseudohypoxic drive has the most supportive evidence based on both clinical and basic investigations. Pseudohypoxic drive refers to the activation of hypoxia response signalling pathways under normal oxygen conditions. The concept of pseudohypoxia is best understood in the context of the VHL pathway.

## THE VHL PATHWAY AND HIF DYSREGULATION

**Figure 1:**
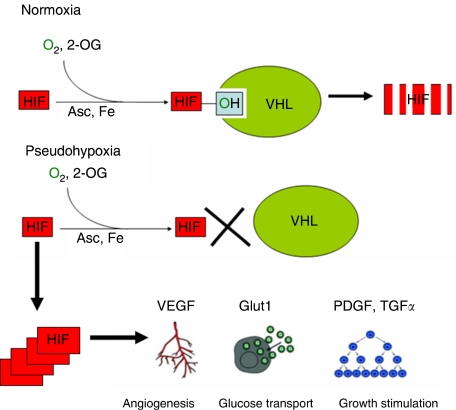
HIF and the VHL complex. Under normoxic conditions, HIF is hydroxylated by HPH. Hypoxia inducible factor 1*α* prolyl hydroxylases activity requires molecular oxygen, 2-OG, ascorbate (Asc), and iron (Fe). Following hydroxylation (represented by the OH group), HIF is recognised by the VHL complex and targeted for degradation. Pseudohypoxia results from *VHL* alterations that result in HIF accumulation with upregulation of potentially tumorigenic genes.

*VHL* is a tumour suppressor gene involved in the development of clear cell RCC. The *VHL* protein product, hereafter referred to as pVHL, is part of a complex signalling cascade that modulates a cell's oxygen-dependent gene expression. In normoxic conditions, pVHL is known to associate with several proteins including Cul-2, Elongin B, and Elongin C to form the VHL complex (Figure 1) (Kibel *et al*, 1995; Linehan *et al*, 2003). Intact VHL complex possesses ubiquitin ligase activity that targets proteins for degradation (Pause *et al*, 1997). Hypoxia inducible factor 1*α* (HIF-1*α*) and HIF-2*α* are two of the proteins targeted for degradation by the VHL complex under normoxic conditions (Ivan *et al*, 2001; Kaelin Jr, 2005). These two proteins are key regulators of oxygen homeostasis (Kaelin Jr, 2005). They are transcription factors involved in the expression of genes involved in nutrient catabolism, angiogenesis, as well as cell growth and differentiation (Semenza, 1999). Target genes of the HIF transcription factors include *vascular endothelial growth factor* (*VEGF*), *glucose transporter 1* (*GLUT1*), *platelet-derived growth factor* (*PDGF*), *transforming growth factor-α* (*TGF*-*α*), and *erythropoietin* (*EPO*). VHL complex recognition of HIF requires the hydroxylation of conserved proline residues of the HIF protein (Ivan *et al*, 2001). Hydroxylation of HIF is carried out by a family of enzymes referred to as HIF prolyl hydroxylases (HPHs), also referred to as prolyl hydroxylases (Kaelin, 2005). The enzymatic hydroxylation of HIF requires molecular oxygen and 2-oxoglutarate (2-OG) as cosubstrates (Kaelin Jr, 2005). In addition, ascorbate and iron are required cofactors for HPH catalytic activity (Kaelin Jr, 2005). Under hypoxic conditions, molecular oxygen is limited. As a result, HIF remains unhydroxylated. In the unhydroxylated state, HIF avoids ubiquitination by the VHL complex, and is therefore available to activate the transcription of the aforementioned genes. In instances where *VHL* mutations are present, HIF is able to avoid degradation, thus creating a pseudohypoxic state. Indeed, upregulation of both HIF-1*α* and HIF-2*α* has been identified in clear cell RCCs along with concomitant expression of HIF downstream target genes (Wiesener *et al*, 2001; Zhang *et al*, 2006). Yet there may be differential contribution of the HIF proteins to tumorigensis. This is in part supported by the contrasting findings in the RCC cell line 786-O, which lacks pVHL that are tumorigenic in mouse xenograft models. Reintroduction of pVHL diminishes tumour formation in xenograft models consistent with the tumour suppressor function of pVHL (Iliopoulos *et al*, 1995). However, the tumour supressor function of pVHL is mitigate in xenograft models with introduction of an HIF-2*α* mutant that avoids ubiquitin-mediated degradation (Kondo *et al*, 2002). On the other hand, the tumour-suppressor function of pVHL remains intact despite the presence of the corresponding HIF-1*α* mutant (Maranchie *et al*, 2002). These and other studies have contributed to the current belief that HIF-2*α* may be more important to the formation of clear cell RCC.

## HLRCC AND PSEUDOHYPOXIC DRIVE

Several reports provide evidence of HIF accumulation in both leiomyomas and renal tumours from HLRCC individuals. Concurrent studies in HPGL have also implicated pseudohypoxic drive and by analogy support involvement of pseudohypoxia in the aetiology of HLRCC kidney tumours. Given that both FH and SDH represent TCA cycle enzymes, it seems reasonable to consider the possibility that their inactivation may uncover a similar mechanism linking mitochondrial dysfunction with oncogenesis.

The notion of TCA cycle dysregulation and pseudohypoxic drive was initially derived from studies in paraganglioma. The increased frequency of carotid body paragangliomas in individuals who resided in higher altitudes (Pacheco-Ojeda *et al*, 1988) suggested a role for chronic hypoxia (Gimenez-Roqueplo *et al*, 2001). The similarity between HPGL tumours from patients with germline *SDHD* mutations and normal carotid body tissue exposed to chronic hypoxia led Baysal *et al* (2000) to suggest that *SDHD* was a critical gene involved in oxygen sensing. Furthermore, they proposed that loss of *SDHD* would lead to what they referred to as ‘hypoxic stimulation’ and cellular proliferation. Tumours from HPGL patients with *SDHD* mutations revealed enhanced expression of HIF as well as VEGF (Gimenez-Roqueplo *et al*, 2001). These and other studies helped formulate the rationale for evaluating the role of pseudohypoxic drive in HLRCC.

Pollard *et al* (2005b) examined HIF-1*α* expression in kidney tumours (both papillary and collecting duct histologies) in patients with HLRCC. Their findings in kidney tumours were compelling for activation of hypoxic pathways. In all renal tumours examined, they noted strong HIF-1*α* staining in the nuclei of tumour cells. This finding was also confirmed by immunoblotting, as HIF-1*α* was easily detected in tumour cell lysate with no detectable HIF-1*α* in normal kidney lysate (Pollard *et al*, 2005b). Isaacs *et al* (2005) also examined HIF-1*α* expression in kidney tumours from patients with HLRCC. Interestingly, six of the seven tumours were from patients less than 40 years of age. HIF expression, evaluated by immunohistochemisty, was determined in cancerous tissues in relation to normal, matched renal parenchyma from the same patient. Both HIF-1*α* and HIF-2*α* were found to be significantly overexpressed in HLRCC renal tumours, but HIF-1*α* expression seemed to be preferentially increased compared to HIF-2*α*.

Further supportive evidence comes from observation of the upregulated downstream HIF targets in tumour tissues isolated from patients with HLRCC. Pollard *et al* (2005a) examined microvessel density in HLRCC uterine leiomyomas. Vascular density, determined by immunohistochemical detection of the vascular endothelial markers, was significantly higher in HLRCC uterine leiomyomas as compared to non-leiomyomatous myometrium from HLRCC women. Furthermore, there was a statistically significant higher vascular density in HLRCC leiomyomas compared to sporadic leiomyomas or normal myometrium procured from women without HLRCC (Pollard *et al*, 2005a). Interestingly, sporadic uterine leiomyomas actually had diminished vascular density as compared to normal myometrium controls from matched patients. In concordance with this finding, *in situ* hybridisation studies revealed upregulation of *VEGF* transcripts in HLRCC uterine leiomyomas in comparison to normal myometrium (from HLRCC and non-HLRCC women) as well as sporadic uterine leiomyomas (Pollard *et al*, 2005a). These findings were confirmed by quantitative real-time PCR data that revealed enhanced expression of *VEGF* (1.4–3.5-fold) as compared to patient-matched normal myometrium. In addition, other hypoxia-responsive gene changes were also found in HLRCC leiomyomas including downregulation of *TSP1*, a known antiangiogenesis factor (Pollard *et al*, 2005a). This investigation suggests significant differences between sporadic leiomyomas and HLRCC leiomyomas with regard to mechanism of tumorigenesis. Indeed, complementary evidence for different mechanisms is indicated by the finding that biallelic *FH* alterations are found in an extremely small subset of sporadic uterine leiomyomas (Lehtonen *et al*, 2004). Further investigation subsequently revealed moderate HIF-1*α* expression in HLRCC fibroids with weak/moderate staining of surrounding myometrium (Pollard *et al*, 2005b). Although overexpression of HIF-1*α* in HLRCC fibroids is less than VEGF, the concomitant upregulation of these proteins suggests that pseudohypoxic activation contributes to the genesis of uterine leiomyomas in HLRCC patients. There is also supportive evidence for activation of hypoxic pathways in kidney tumours from HLRCC patients. Isaacs *et al* (2005) identified enhanced GLUT1 expression by immunohistochemisty in multiple HLRCC kidney tumours as compared to normal renal tissue.

Although there is clear evidence of activation of pseudohypoxic pathways in HLRCC tumours, the molecular mechanism underlying this phenomenon remains obscure. Unlike the involvement of pVHL in HIF degradation, there is no direct link between the FH and HIF proteins. The answer may lie in the biochemical alterations that result from the absence of an intact FH enzyme. As noted earlier, FH catalyses the hydration of fumarate to form malate. The absence of FH could presumably result in chronically elevated levels of fumarate and altered levels of other TCA intermediates and molecules indirectly linked to the TCA cycle. There is direct evidence to support elevation of intracellular fumarate secondary to reduced FH activity as a proximal cause of pseudohypoxia. Isaacs *et al* (2005) transfected the FH wild-type lung carcinoma cell line A549 with small interfering RNA (siRNA) targeting FH. The intracellular fumarate level rapidly doubled in treated A549 cells. Although these data support the possibility that reduced *FH* expression may acutely result in elevated intracellular fumarate, they may not be representative of cellular changes associated with long-term absence of *FH* expression. However, Pollard *et al* (2005b) addressed this issue by examining levels of various Kreb's cycle intermediates in uterine leiomyomas from HLRCC patients. Fumarate levels in these HLRCC fibroids were markedly elevated (over 200-fold) as compared to levels in normal myometrium from both non-HLRCC and HLRCC patients. Interestingly, succinate levels were also found to be elevated in HLRCC fibroids, but to a lesser degree than fumarate. Although similar analysis of HLRCC kidney tumours does not yet exist, these findings certainly support the possibility that elevated fumarate levels may represent a chronic cellular response to impaired/absent FH activity.

**Figure 2:**
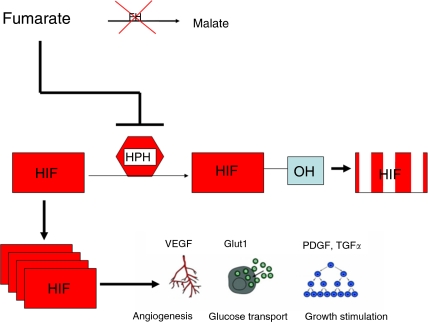
Hereditary leiomyomatosis and renal cell cancer pseudohypoxia. Fumarate accumulates due to loss of FH activity. Rising fumarate levels inhibit HPH with concomitant upregulation of hypoxia response genes including *VEGF* and *GLUT-1*.

Given the likelihood that cells with impaired/absent FH activity have elevated levels of fumarate, the question remains as to why this results in a cellular milieu prone to tumorigenesis. Isaacs *et al* (2005) examined the potential link between fumarate and HIF stability utilising a known inhibitor of both FH and SDH, 3-nitropropionic acid (Coles *et al*, 1979; Porter and Bright, 1980). In A549 lung carcinoma cells, inhibition of FH activity in addition to exogenous administration of fumarate resulted in elevated HIF levels in nuclear protein extracts. Furthermore, similar experiments performed in the presence of cycloheximide, a protein synthesis inhibitor, revealed that the increased level of HIF was due to increased HIF protein half-life (approximately five-fold longer than control treatment). Combination treatment of FH inhibitor and fumarate resulted in a diminished level of hydroxylated HIF. As noted earlier, hydroxylated HIF is the form of HIF that is recognisable by the VHL complex for degradation. Given these data, Isaacs *et al* (2005) further examined the effect of fumarate on HPH-dependent HIF hydroxylation, and they found that the ratio of hydroxylated HIF to total HIF protein was dramatically reduced by pharmacologic elevation of intracellular fumarate level. As noted earlier, HIF degradation relies on the hydroxylation of proline residues by the HPH enzymes, requiring molecular oxygen and 2-OG as cosubstrates. In cell-free systems, Isaacs *et al* (2005) found fumarate to be a potent competitive inhibitor of the HPH cosubstrate 2-OG (Figure 2). Given evidence of fumarate elevation in *FH* null tumours, these findings provide a clear rationale for pseudohypoxic drive in HLRCC being HIF-dependent.

Further supporting the link between Kreb's cycle dysfunction and pseudohypoxic drive are previous studies investigating the role of *SDH* mutations in HPGL. Indeed the findings of these prior reports indicate parallel mechanisms between succinate and fumarate-induced pseudohypoxic drive as succinate may interfere with HPH activity. As a by-product of HIF hydroxylation by HPH, the cosubstrate 2-OG undergoes oxidative decarboxylation to form succinate (Gottlieb and Tomlinson, 2005). Selak *et al* (2005) demonstrated that inhibition of SDH with siRNA resulted in elevated HIF levels owing to inhibition of HPH. Furthermore, these investigators demonstrated that succinate is able to inhibit HPH directly, most likely via product feedback inhibition (Selak *et al*, 2005). Pollard *et al* (2005b) demonstrated strong nuclear HIF-1*α* expression in tumours (both paragangliomas and pheochromocytomas) from HPGL patients. Paragangliomas from patients with known germline *SDHB* mutations have been found to have elevated succinate levels (Pollard *et al*, 2005b). In addition, multiple reports have reported enhanced expression of HIF-regulated genes including *VEGF* in tumours with known *SDH* mutations (Gimenez-Roqueplo *et al*, 2001; Pollard *et al*, 2005a). Although these studies focus on the effects of an acute change in SDH activity, paragangliomas from patients with germline *SDHB* mutations have been shown to have chronically elevated levels of succinate (Pollard *et al*, 2005b). In concordance with this finding is a recent report indicating that *SDHA* mutant fibroblasts accumulate succinate and that succinate facilitates nuclear translocation of HIF (Briere *et al*, 2005).

Taken together, these data provide compelling evidence for a link between Kreb's cycle dysfunction and oncogenesis. Despite possibly sharing a similar mechanism for tumorigenesis, HLRCC and HPGL represent two distinct syndromes with minimal overlap. There are no reported cases of pheochromocytoma in patients with known germline *FH* mutations. However, there is one report of early onset RCC in three patients with germline *SDHB* mutations (Vanharanta *et al*, 2004). Two of the three patients were from the same family and all three RCCs were found to have lost the wild-type *SDHB* allele. This small data set provides further evidence that TCA cycle dysregulation plays an important role in RCC aetiology.

## CONCLUSION

Hereditary leiomyomatosis and renal cell cancer is a hereditary cancer syndrome predisposing individuals to the development of aggressive kidney cancers. These individuals are known to harbour a germline mutation of *FH*, which codes for the enzyme that catalyses the conversion of fumarate to malate. Although multiple mechanisms of tumorigenesis have been proposed, the preponderance of data thus far supports a role for pseudohypoxic drive involving fumarate-dependent HIF activation. The link between FH inactivation and HIF appears to involve inhibition of HPH activity (and thus pVHL recognition of HIF) via fumarate accumulation. By a similar mechanism, elevated levels of succinate may be responsible for pseudohypoxic drive in the context of paragangliomas/pheochromocytomas in patients with germline *SDH* mutations. These studies provide a unique molecular basis for the Warburg effect. The Warburg effect refers to the preferential derivation of energy by rapidly dividing cancer cells from glucose metabolism via glycolysis rather than oxidative phosphorylation despite adequate intracellular oxygen levels. We have shown that experimental depletion of FH results in acute upregulation of GLUT1 protein, glucose uptake, and lactate production in normoxic cells (Isaacs *et al*, 2005). Succinate accumulation may produce a similar phenotype (Selak *et al*, 2005). Lu *et al* (2005) have proposed that various glucose metabolites promote HIF stabilisation and reduced expression of TCA cycle enzymes, thus constituting a feed-forward signalling mechanism favouring continued dependence on glycolysis as an energy source. This dependence is further driven by growth factors frequently associated with oncogenesis (e.g., insulin, insulin-like growth factor-1, epidermal growth factor, heregulin) that stimulate the AKT–mTOR pathway and subsequently the rate of HIF translation (Majumder *et al*, 2004). Thus, although the molecular mechanisms responsible for this metabolic switch are complex and likely multifactorial, the ultimate upregulation of HIF may provide a cellular milieu supportive of continuously elevated glycolysis and suppressed oxidative phosphorylation. Continued investigation of the molecular mechanisms underlying pseudohypoxia will therefore elucidate new targets for molecular therapeutics that are applicable to both inherited and sporadic kidney cancers.

## References

[bib1] Astuti D, Latif F, Dallol A, Dahia PL, Douglas F, George E, Skoldberg F, Husebye ES, Eng C, Maher ER (2001) Gene mutations in the succinate dehydrogenase subunit SDHB cause susceptibility to familial pheochromocytoma and to familial paraganglioma. Am J Hum Genet 69: 49–54 11404820 10.1086/321282 PMC1226047

[bib2] Baysal BE, Ferrell RE, Willett-Brozick JE, Lawrence EC, Myssiorek D, Bosch A, van der MA, Taschner PE, Rubinstein WS, Myers EN, Richard III CW, Cornelisse CJ, Devilee P, Devlin B (2000) Mutations in SDHD, a mitochondrial complex II gene, in hereditary paraganglioma. Science 287: 848–851 10657297 10.1126/science.287.5454.848

[bib3] Briere JJ, Favier J, Benit P, El GV, Lorenzato A, Rabier D, Di Renzo MF, Gimenez-Roqueplo AP, Rustin P (2005) Mitochondrial succinate is instrumental for HIF1alpha nuclear translocation in SDHA-mutant fibroblasts under normoxic conditions. Hum Mol Genet 14: 3263–3269 16195397 10.1093/hmg/ddi359

[bib4] Coles CJ, Edmondson DE, Singer TP (1979) Inactivation of succinate dehydrogenase by 3-nitropropionate. J Biol Chem 254: 5161–5167 447637

[bib5] Gimenez-Roqueplo AP, Favier J, Rustin P, Mourad JJ, Plouin PF, Corvol P, Rotig A, Jeunemaitre X (2001) The R22X mutation of the SDHD gene in hereditary paraganglioma abolishes the enzymatic activity of complex II in the mitochondrial respiratory chain and activates the hypoxia pathway. Am J Hum Genet 69: 1186–1197 11605159 10.1086/324413 PMC1235531

[bib6] Gnarra JR, Tory K, Weng Y, Schmidt L, Wei MH, Li H, Latif F, Liu S, Chen F, Duh FM (1994) Mutations of the VHL tumour suppressor gene in renal carcinoma. Nat Genet 7: 85–90 7915601 10.1038/ng0594-85

[bib7] Gottlieb E, Tomlinson IP (2005) Mitochondrial tumour suppressors: a genetic and biochemical update. Nat Rev Cancer 5: 857–866 16327764 10.1038/nrc1737

[bib8] Iliopoulos O, Kibel A, Gray S, Kaelin Jr WG (1995) Tumour suppression by the human von Hippel–Lindau gene product. Nat Med 1: 822–826 7585187 10.1038/nm0895-822

[bib9] Isaacs JS, Jung YJ, Mole DR, Lee S, Torres-Cabala C, Chung YL, Merino M, Trepel J, Zbar B, Toro J, Ratcliffe PJ, Linehan WM, Neckers L (2005) HIF overexpression correlates with biallelic loss of fumarate hydratase in renal cancer: novel role of fumarate in regulation of HIF stability. Cancer Cell 8: 143–153 16098467 10.1016/j.ccr.2005.06.017

[bib10] Ivan M, Kondo K, Yang H, Kim W, Valiando J, Ohh M, Salic A, Asara JM, Lane WS, Kaelin Jr WG (2001) HIFalpha targeted for VHL-mediated destruction by proline hydroxylation: implications for O2 sensing. Science 292: 464–468 11292862 10.1126/science.1059817

[bib11] Kaelin WG (2005) Proline hydroxylation and gene expression. Annu Rev Biochem 74: 115–128 15952883 10.1146/annurev.biochem.74.082803.133142

[bib12] Kaelin Jr WG (2005) The von Hippel–Lindau protein, HIF hydroxylation, and oxygen sensing. Biochem Biophys Res Commun 338: 627–638 16153592 10.1016/j.bbrc.2005.08.165

[bib13] Kibel A, Iliopoulos O, DeCaprio JA, Kaelin Jr WG (1995) Binding of the von Hippel–Lindau tumor suppressor protein to Elongin B and C. Science 269: 1444–1446 7660130 10.1126/science.7660130

[bib14] Kiuru M, Lehtonen R, Arola J, Salovaara R, Jarvinen H, Aittomaki K, Sjoberg J, Visakorpi T, Knuutila S, Isola J, Delahunt B, Herva R, Launonen V, Karhu A, Aaltonen LA (2002) Few FH mutations in sporadic counterparts of tumor types observed in hereditary leiomyomatosis and renal cell cancer families. Cancer Res 62: 4554–4557 12183404

[bib15] Kondo K, Klco J, Nakamura E, Lechpammer M, Kaelin Jr WG (2002) Inhibition of HIF is necessary for tumor suppression by the von Hippel–Lindau protein. Cancer Cell 1: 237–246 12086860 10.1016/s1535-6108(02)00043-0

[bib16] Latif F, Tory K, Gnarra J, Yao M, Duh FM, Orcutt ML, Stackhouse T, Kuzmin I, Modi W, Geil L (1993) Identification of the von Hippel–Lindau disease tumor suppressor gene. Science 260: 1317–1320 8493574 10.1126/science.8493574

[bib17] Launonen V, Vierimaa O, Kiuru M, Isola J, Roth S, Pukkala E, Sistonen P, Herva R, Aaltonen LA (2001) Inherited susceptibility to uterine leiomyomas and renal cell cancer. Proc Natl Acad Sci USA 98: 3387–3392 11248088 10.1073/pnas.051633798 PMC30663

[bib18] Lehtonen R, Kiuru M, Vanharanta S, Sjoberg J, Aaltonen LM, Aittomaki K, Arola J, Butzow R, Eng C, Husgafvel-Pursiainen K, Isola J, Jarvinen H, Koivisto P, Mecklin JP, Peltomaki P, Salovaara R, Wasenius VM, Karhu A, Launonen V, Nupponen NN, Aaltonen LA (2004) Biallelic inactivation of fumarate hydratase (FH) occurs in nonsyndromic uterine leiomyomas but is rare in other tumors. Am J Pathol 164: 17–22 14695314 10.1016/S0002-9440(10)63091-X PMC1602244

[bib19] Linehan WM, Walther MM, Zbar B (2003) The genetic basis of cancer of the kidney. J Urol 170: 2163–2172 14634372 10.1097/01.ju.0000096060.92397.ed

[bib20] Lu H, Dalgard CL, Mohyeldin A, McFate T, Tait AS, Verma A (2005) Reversible inactivation of HIF-1 prolyl hydroxylases allows cell metabolism to control basal HIF-1. J Biol Chem 280: 41928–41939 16223732 10.1074/jbc.M508718200

[bib21] Majumder PK, Febbo PG, Bikoff R, Berger R, Xue Q, McMahon LM, Manola J, Brugarolas J, McDonnell TJ, Golub TR, Loda M, Lane HA, Sellers WR (2004) mTOR inhibition reverses Akt-dependent prostate intraepithelial neoplasia through regulation of apoptotic and HIF-1-dependent pathways. Nat Med 10: 594–601 15156201 10.1038/nm1052

[bib22] Maranchie JK, Vasselli JR, Riss J, Bonifacino JS, Linehan WM, Klausner RD (2002) The contribution of VHL substrate binding and HIF1-alpha to the phenotype of VHL loss in renal cell carcinoma. Cancer Cell 1: 247–255 12086861 10.1016/s1535-6108(02)00044-2

[bib23] Morris MR, Maina E, Morgan NV, Gentle D, Astuti D, Moch H, Kishida T, Yao M, Schraml P, Richards FM, Latif F, Maher ER (2004) Molecular genetic analysis of FIH-1, FH, and SDHB candidate tumour suppressor genes in renal cell carcinoma. J Clin Pathol 57: 706–711 15220362 10.1136/jcp.2003.011767 PMC1770369

[bib24] Niemann S, Muller U (2000) Mutations in SDHC cause autosomal dominant paraganglioma, type 3. Nat Genet 26: 268–270 11062460 10.1038/81551

[bib25] Pacheco-Ojeda L, Durango E, Rodriquez C, Vivar N (1988) Carotid body tumors at high altitudes: Quito, Ecuador, 1987. World J Surg 12: 856–860 3250136 10.1007/BF01655498

[bib26] Pause A, Lee S, Worrell RA, Chen DY, Burgess WH, Linehan WM, Klausner RD (1997) The von Hippel–Lindau tumor-suppressor gene product forms a stable complex with human CUL-2, a member of the Cdc53 family of proteins. Proc Natl Acad Sci USA 94: 2156–2161 9122164 10.1073/pnas.94.6.2156 PMC20057

[bib27] Pollard P, Wortham N, Barclay E, Alam A, Elia G, Manek S, Poulsom R, Tomlinson I (2005a) Evidence of increased microvessel density and activation of the hypoxia pathway in tumours from the hereditary leiomyomatosis and renal cell cancer syndrome. J Pathol 205: 41–49 15586379 10.1002/path.1686

[bib28] Pollard PJ, Briere JJ, Alam NA, Barwell J, Barclay E, Wortham NC, Hunt T, Mitchell M, Olpin S, Moat SJ, Hargreaves IP, Heales SJ, Chung YL, Griffiths JR, Dalgleish A, McGrath JA, Gleeson MJ, Hodgson SV, Poulsom R, Rustin P, Tomlinson IP (2005b) Accumulation of Krebs cycle intermediates and over-expression of HIF1alpha in tumours which result from germline FH and SDH mutations. Hum Mol Genet 14: 2231–2239 15987702 10.1093/hmg/ddi227

[bib29] Porter DJ, Bright HJ (1980) 3-Carbanionic substrate analogues bind very tightly to fumarase and aspartase. J Biol Chem 255: 4772–4780 7372610

[bib30] Schmidt L, Duh FM, Chen F (1997) Germline and somatic mutations in the tyrosine kinase domain of the MET proto-oncogene in papillary renal carcinomas. Nat Genet 16: 68–73 9140397 10.1038/ng0597-68

[bib31] Selak MA, Armour SM, MacKenzie ED, Boulahbel H, Watson DG, Mansfield KD, Pan Y, Simon MC, Thompson CB, Gottlieb E (2005) Succinate links TCA cycle dysfunction to oncogenesis by inhibiting HIF-alpha prolyl hydroxylase. Cancer Cell 7: 77–85 15652751 10.1016/j.ccr.2004.11.022

[bib32] Semenza GL (1999) Regulation of mammalian O_2_ homeostasis by hypoxia-inducible factor 1. Annu Rev Cell Dev Biol 15: 551–578 10611972 10.1146/annurev.cellbio.15.1.551

[bib33] Tomlinson IP, Alam NA, Rowan AJ (2002) Germline mutations in FH predispose to dominantly inherited uterine fibroids, skin leiomyomata and papillary renal cell cancer. Nat Genet 30: 406–410 11865300 10.1038/ng849

[bib34] Toro JR, Nickerson ML, Wei MH, Warren MB, Glenn GM, Turner ML, Stewart L, Duray P, Tourre O, Sharma N, Choyke P, Stratton P, Merino M, Walther MM, Linehan WM, Schmidt LS, Zbar B (2003) Mutations in the fumarate hydratase gene cause hereditary leiomyomatosis and renal cell cancer in families in North America. Am J Hum Genet 73: 95–106 12772087 10.1086/376435 PMC1180594

[bib35] Vanharanta S, Buchta M, McWhinney SR, Virta SK, Peczkowska M, Morrison CD, Lehtonen R, Januszewicz A, Jarvinen H, Juhola M, Mecklin JP, Pukkala E, Herva R, Kiuru M, Nupponen NN, Aaltonen LA, Neumann HP, Eng C (2004) Early-onset renal cell carcinoma as a novel extraparaganglial component of SDHB-associated heritable paraganglioma. Am J Hum Genet 74: 153–159 14685938 10.1086/381054 PMC1181902

[bib36] Wei MH, Toure O, Glenn GM, Pithukpakorn M, Neckers L, Stolle C, Choyke P, Grubb R, Middelton L, Turner ML, Walther MM, Merino MJ, Zbar B, Linehan WM, Toro JR (2006) Novel mutations in FH and expansion of the spectrum of phenotypes expressed in families with hereditary leiomyomatosis and renal cell cancer. J Med Genet 43: 18–27 15937070 10.1136/jmg.2005.033506 PMC2564499

[bib37] Wiesener MS, Munchenhagen PM, Berger I, Morgan NV, Roigas J, Schwiertz A, Jurgensen JS, Gruber G, Maxwell PH, Loning SA, Frei U, Maher ER, Grone HJ, Eckardt KU (2001) Constitutive activation of hypoxia-inducible genes related to overexpression of hypoxia-inducible factor-1alpha in clear cell renal carcinomas. Cancer Res 61: 5215–5222 11431362

[bib38] Zhang N, Gong K, Yang XY, Xin DQ, Na YQ (2006) Expression of hypoxia-inducible factor-1-alpha, hypoxia-inducible factor-2alpha and vascular endothelial growth factor in sporadic clear cell renal cell renal cell carcinoma and their significance in the pathogenesis thereof. Zhonghua Yi Xue Za Zhi 86: 1526–1529 16854277

